# Static Stretching Acutely Reduces the Ergogenic Effects of Caffeine on Sprint Performance but Not Maximal Ball Velocity in Football Players

**DOI:** 10.1002/ejsc.70064

**Published:** 2026-01-21

**Authors:** Refik Çabuk, İzzet İslamoğlu, Onur Demirarar, Faruk Albay, Yıldırım Kayacan

**Affiliations:** ^1^ Department of Coaching Education Yaşar Doğu Faculty of Sports Sciences Ondokuz Mayıs University Samsun Türkiye; ^2^ College of Health and Life Sciences Hamad Bin Khalifa University Doha Qatar; ^3^ Department of Physical Education and Sports Sciences Gendarmerie and Coast Guard Academy Ankara Türkiye; ^4^ Department of Sport Management Yaşar Doğu Faculty of Sports Sciences Ondokuz Mayıs University Samsun Türkiye

**Keywords:** anaerobic power, ball velocity, ergogenic effect, explosive power, instep kicking

## Abstract

This study aimed to investigate the independent and combined effects of caffeine and static stretching (SS) on maximal ball velocity and 30‐m sprint performance. Sixteen male amateur football players performed 30‐m sprint and an instep kicking ball velocity test under six conditions. The six conditions were control, SS only, placebo only, placebo combined with SS (PLA + SS), caffeine only, and caffeine combined with SS (CAF + SS). A repeated measures ANOVA revealed no significant main effect of condition on maximal ball velocity (*F*(5, 75) = 1.11, *p* = 0.36, *η* ^2^_p_ = 0.069). In contrast, a significant main effect of condition was observed for 30‐m sprint performance (*F*(5, 75) = 4.57, *p* = 0.001, *η* ^2^_p_ = 0.23). Our findings showed that the SS condition resulted in similar sprint performance compared to all other conditions (*p* ≥ 0.093) except the caffeine condition (*p* = 0.009). In contrast, the caffeine condition led to faster sprint performance compared to all conditions (*p* ≤ 0.010) except CAF + SS (*p* = 0.184). Additionally, sprint performances in the SS and CAF + SS conditions were similar (*p* = 0.093). A large effect size (0.94) was observed between the control and CAF conditions, whereas a moderate effect size (0.54) was found when comparing caffeine and CAF + SS conditions. These findings indicate that caffeine intake enhances sprint performance; however, the CAF + SS combination appears to reduce this effect, making it less effective. Although SS does not directly impair sprint performance, it may have the potential to diminish the ergogenic effects of caffeine.

## Key Highlights

- Caffeine ingestion significantly enhanced 30‐m sprint performance.
- Static stretching alone had no negative effect on the sprint or maximal ball velocity.
- When combined with caffeine, static stretching reduced its ergogenic benefit on sprint performance.
- These findings offer practical guidance on warm‐up planning and ergogenic aid use.

## 1 Introduction

Sports professionals and coaches continuously seek ways to enhance physical performance. One of the most used and extensively researched ergogenic aids among available strategies is caffeine (CAF; Kerksick et al. 2018; Maughan et al. 2018). Current evidence suggests that consuming low‐to‐moderate doses of caffeine (3–6 mg·kg^−1^) approximately 60 min prior to exercise can enhance anaerobic performance (Grgic 2017; Grgic and Mikulic 2017; Acar et al. 2024; A. Mor et al. 2024; H. Mor et al. 2025). Additionally, the International Olympic Committee's consensus statement on dietary supplements identified CAF as one of the few supplements with well‐established performance‐enhancing benefits (Maughan et al. 2018). The ergogenic effects of CAF are attributed to both central and peripheral mechanisms (J. K. Davis and Green 2009; J. M. Davis et al. 2003). Centrally, CAF acts as an adenosine A1 and A2a receptor antagonist, which reduces fatigue, enhances neurotransmission, and increases motor unit firing rates (J. M. Davis et al. 2003; Tallis et al. 2015). Peripherally, CAF supports muscle contraction by promoting calcium ion mobilization (Rousseau et al. 1988) and exerts its effects through phosphodiesterase inhibition (J. K. Davis and Green 2009). Through these mechanisms, CAF contributes to improvements in muscular strength and explosive power, making it an ideal supplement for athletic performance.

Although the ergogenic effects of CAF are well‐documented when used in isolation (Kerksick et al. 2018; Grgic 2017; Grgic and Mikulic 2017; Maughan et al. 2018; Waer et al. 2024), recent studies have increasingly focused on combining CAF with other ergogenic strategies to enhance its performance benefits (Abdioğlu et al. 2025; Çabuk et al. 2025; Filip‐Stachnik et al. 2022; Suksuwan et al. 2022). In fact, combining CAF with other supplements (e.g., sodium bicarbonate, carbohydrates; Suksuwan et al. 2022; Felippe et al. 2016), training strategies (e.g., conditioning activities during warm‐up; Ouergui et al. 2022), or psycho‐affective stimuli (e.g., music; Delleli et al. 2023, 2024) has been shown to produce synergistic effects. These ergogenic strategies are generally known to have acute performance‐enhancing potential even when used alone (Delleli et al. 2023, 2024; Guerra et al. 2018; Impey et al. 2022) or, at the very least, do not impair performance (de Oliveira et al. 2020; Filip‐Stachnik et al. 2022). However, although many of these strategies, when combined with CAF, do not influence strength‐related tasks (Wang et al. 2016; Filip‐Stachnik et al. 2022), the effects of static stretching (SS), which has been reported to negatively impact strength‐ and speed‐related tasks in some studies (Behm et al. 2016; Gelen 2010; Amiri‐Khorasani and Ferdinands 2014), remain unclear when combined with CAF (Çabuk et al. 2025). Behm et al. (2016) reported a small overall performance decrement of approximately 1.3% in strength‐ and speed‐related tasks following SS. They concluded that this reduction was not severe enough to warrant the complete removal of SS from warm‐up protocols. However, in individual sports such as sprinting, long jump, high jump, shot put, or javelin throw, even such small decrements may have practical significance. Moreover, these minor differences may also play a critical role in team sports where anaerobic performance is a key determinant of success. On the other hand, for athletes who have adopted SS as a habitual component of their warm‐up routines or strongly believe in its necessity, removing it could have negative psychological effects (Young 2007). These beliefs and habits may explain why SS continues to be used despite ongoing scientific debate (Popp et al. 2017; Young 2007).

To the best of our knowledge, only two studies have examined the combined effects of CAF and SS on performance outcomes, specifically 1‐repetition maximum (1RM) knee flexion (Farney et al. 2019) and Wingate anaerobic test (Çabuk et al. 2025). Both studies hypothesized that CAF might counteract the potential neural impairments caused by SS through the stimulation of the central nervous system and increased motor unit activation. However, their findings were not consistent. For instance, Farney et al. (2019) concluded that CAF ingestion (6 mg·kg^−1^) did not eliminate the reduction in 1RM knee flexion strength observed following an SS protocol (4 sets of 4 SS exercises × 30 s with 15 s rest). Regardless of whether participants consumed CAF or placebo (PLA), they experienced an approximately 7.5% decrease in performance after SS compared to the no‐stretching condition. Although their study did not directly focus on the interaction between SS and CAF's ergogenic effects, the results suggest that SS may attenuate the performance‐enhancing effects of CAF. In contrast, Çabuk et al. (2025) found that, under CAF conditions (6 mg·kg^−1^), the performance decrements caused by SS (2 sets of 9 SS exercises × 30 s with 10 s rest) were mitigated during the Wingate anaerobic test, specifically in peak power and average power outputs, when compared to the static and PLA + SS conditions. Nevertheless, they reported that SS still partially diminished the ergogenic impact of CAF. Given that only two studies have explored this topic (Çabuk et al. 2025; Farney et al. 2019), it is premature to draw definitive conclusions regarding the interactive effects of CAF and SS on performance. Therefore, additional placebo‐controlled studies using various participant groups and sport‐specific field performance tests are warranted. Building on this background, the current study aims to investigate the independent and combined effects of CAF and SS on maximal ball velocity and 30‐m sprint performance on amateur football players.

## 2 Methods

### 2.1 Experimental Design

This study employed a randomized, double‐blind, placebo‐controlled crossover design to determine the actual and perceived effects of SS, CAF ingestion, and their combination on performance. Each participant completed six separate laboratory visits. To minimize training adaptations and control for circadian rhythm effects, all sessions were scheduled within a 21‐day period and conducted at the same time of day (± 1 h). There was a minimum of 72 h between visits. All tests were conducted in a temperature‐controlled environment (22°C–23°C).

**FIGURE 1:**
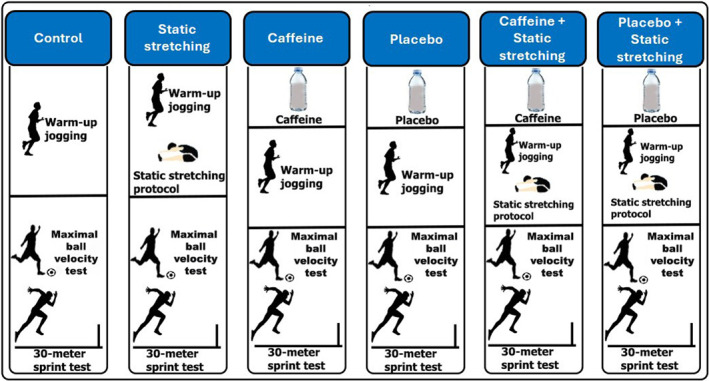
This figure illustrates the six experimental conditions used in the randomized, double‐blind, placebo‐controlled crossover study designed to investigate the effects of static stretching, caffeine ingestion, and their combination on maximal ball velocity and sprint performance.

As part of the standardized warm‐up, participants performed 5 minutes of light jogging. Following the warm‐up, participants completed one of six experimental conditions, after which maximal ball velocity and 30‐m sprint performance were assessed. The six conditions were control (CON), SS only, PLA only, PLA combined with SS (PLA + SS), CAF only, and CAF combined with SS (CAF + SS). The experimental design of the study is presented in Figure 1. Participants ingested either powdered caffeine or placebo (all‐purpose flour) dissolved in 300 mL of water, 60 min before the performance tests (see caffeine and placebo intake section for further details).

### 2.2 Participants

An a priori power analysis was conducted using G*Power software (*F*‐test) to determine the required sample size. Based on an effect size (*f*) of 0.50, an alpha level (*α*) of 0.05, statistical power (1−*β*) of 0.95, one group, and six repeated measures, the minimum required sample size was calculated as eight. However, in the present study, a total of 16 participants were recruited to increase the reliability of the findings and to account for potential data loss, which exceeded the minimum required sample size. Therefore, 16 male amateur football players (age: 22.2 ± 2.4 years; height: 179 ± 4.6 cm; body mass: 70.7 ± 9.7 kg) volunteered to participate in this study. All participants had comparable training backgrounds, with an average of 6.7 ± 1.8 years of continuous football training experience and regular participation in amateur‐level competitions. On average, they trained 4.3 ± 0.5 sessions per week (range: 4–5 sessions). None reported musculoskeletal injuries in the 6 months preceding the study. The study was approved by the Institutional Review Board of the Gendarmerie and Coast Guard Academy (Ethics approval number: E‐97703210‐050.99‐37067511) and conducted in accordance with the Declaration of Helsinki. Prior to providing written informed consent, participants were fully informed about the nature and potential risks of the study.

The inclusion criteria required participants to be male amateur football players aged 18 years or older, actively competing in league matches, and habitually less than ∼1 mg·kg^−1^ of caffeine per day. Individuals who experienced adverse effects from caffeine during the study were excluded. To assist participants in estimating their intake, detailed information regarding common caffeine‐containing foods and their approximate amounts was provided. Throughout the testing period, participants were instructed to abstain from alcohol, any additional caffeine intake or supplementation, and other ergogenic aids to avoid potential interactions with caffeine. Apart from these restrictions, participants were asked to maintain their habitual diets. In addition, they were specifically instructed to refrain from caffeine‐containing foods and strenuous exercise for 24 h prior to each testing session. To minimize potential training adaptations, the time between the first and the last testing session (a total of six sessions) did not exceed 3 weeks for any participant.

### 2.3 Procedures

#### 2.3.1 Static Stretching Protocol

One minute after completing the warm‐up protocol, participants completed nine lower‐body SS exercises. These included stretches for the (1) gastrocnemius, (2) tibialis anterior, (3) hamstring muscles, (4) quadriceps, (5) gluteus maximus, (6) iliopsoas, (7) hip adductors, (8) hip abductors, and (9) quadratus lumborum. Exercises 1 and 4–9 were performed separately for both the right and left legs. Participants were instructed to hold each stretch for 30 s, based on their individual perceived stretch intensity, reaching their personal discomfort threshold. Each SS exercise was separated by a 10‐s passive rest period and was performed twice, totaling 60 s of stretching per muscle/muscle group. The total duration of the SS protocol was approximately 22 min. All stretches were self‐administered without assistance. Three minutes after completing the static stretching protocol, participants performed the maximal ball velocity test.

#### 2.3.2 Caffeine and Placebo Intake

CAF typically reaches peak plasma concentration approximately 45–60 min after ingestion (Sökmen et al. 2008). Therefore, participants consumed either a CAF or PLA solution 60 min before the performance tests, dissolved in 300 mL of water. In accordance with NCAA recommendations, the CAF dose was set at 6 mg·kg^−1^ body mass. Both CAF and PLA (all‐purpose flour) were prepared in powder form, with CAF dosages measured using an electronic scale accurate to 0.001 g (Kern ABJ‐NM/ABS‐N, Kern & Sohn GmbH, Germany). To prevent expectancy effects related to appearance or taste, the beverages were matched for volume, color, consistency, and flavor. The naturally bitter taste of CAF was masked with three sweetener tablets, whereas one tablet was added to the PLA drink to provide a comparable flavor profile. In addition, to match the consistency, color, and appearance of the beverages, 1.5 level teaspoons of all‐purpose flour were added to the PLA drink and 0.5 level teaspoon to the CAF drink. This approach minimized potential biases related to taste or appearance.

Participants were informed that a PLA condition would be included in the study, but they were not told the order in which they received CAF or PLA. To assess both the actual and perceived effects of CAF and PLA, participants were asked immediately before testing which condition they believed they had consumed. To ensure an accurate evaluation of the effects of CAF and PLA under blinded conditions, a double‐blind, placebo‐controlled design was used. Although one researcher prepared the beverages, the researchers conducting the performance assessments were blinded to the conditions.

#### 2.3.3 Maximal Ball Velocity in Instep Kicking

Participants were instructed to perform an instep kick with their dominant foot toward a stationary size 5 football (mass: 430 g; pressure: 900 hPa) approved by the Fédération Internationale de Football Association (Adidas, Herzogenaurach, Germany). The ball was placed 11 m from the goal (2 m × 3 m), and players approached the ball from a distance of 2 m. Each participant was given three attempts, with 2‐min rest intervals between trials. They were explicitly instructed to focus solely on generating maximal ball velocity. Any attempts that completely missed the target area were repeated. Ball velocity was measured using a stationary Doppler radar gun (Stalker Sport 2, Stalker Radar, Plano, Texas, USA). The radar was mounted on a 1‐m‐high tripod, positioned behind the goal net and aligned with the center of the goal, approximately 2 m behind the starting position. The highest ball velocity recorded across the three trials was selected for analysis and expressed in km·h^−1^. All testing was conducted indoors in a sports hall with a standard wooden sports floor. Participants wore football‐specific indoor shoes and their regular training apparel. Three minutes after completing the maximal ball velocity test, they performed the linear sprint test.

#### 2.3.4 30‐m Sprint Test

Linear sprint performance was assessed using a 30‐m sprint test. Sprint times were recorded using photoelectric timing gates (Newtest, Finland). The timing gates were mounted on tripods adjusted to a height of 0.9 m and placed in pairs, spaced 1 m apart. Participants began each sprint from a standing start, with the lead foot positioned 0.5 m behind the first timing gate. They were instructed to perform each sprint with maximal effort. Each participant completed three sprints with a 2‐min rest between trials. The fastest time among the three trials was used for analysis. The 30‐m sprint test was conducted indoors on a standard wooden sports floor, under same environmental conditions as the maximal ball velocity test. To avoid variability due to footwear, all participants wore the same football‐specific indoor shoes during both tests.

### 2.4 Statistical Analyses

All data were assessed for normality using the Shapiro–Wilk test. Repeated measures ANOVA was used to analyse the variables, and post hoc comparisons were performed using the least significant difference (LSD) test. Statistical significance was set at *p* < 0.05. Partial eta squared (*η* ^2^_p_) values were calculated for the ANOVA, with effect sizes interpreted as small (< 0.02), medium (0.02–0.06), or large (> 0.14). Additionally, effect sizes (ES) for pairwise comparisons were calculated using Cohen's *d* and interpreted as trivial (< 0.2), small (0.2–0.5), medium (0.5–0.8), or large (> 0.8). The smallest worthwhile change (SWC) was calculated as 0.2 times the standard deviation across all six conditions and was used to evaluate whether CAF and/or SS produced a meaningful positive or negative change in performance compared to the CON condition.

## 3 Results

### 3.1 Repeated Measures ANOVA

A repeated measures ANOVA revealed significant differences in best 30‐m sprint times across the conditions (*F*(5, 75) = 4.57, *p* = 0.001, *η* ^2^_p_ = 0.23). Sprint completion time in the CON condition was significantly slower than in both the CAF (*p* = 0.002; ES = 0.94) and CAF + SS conditions (*p* = 0.046; ES = 0.35). The best sprint time in the CAF condition was significantly faster than in the CON, SS, PLA, and PLA + SS conditions (*p* = 0.009–0.01; ES: 0.74–1.07). Additionally, sprint performance in the CAF + SS condition was significantly better than in the CON, SS, and PLA + SS conditions (*p* = 0.008–0.048; ES: 0.43–0.76) (Table 1).

**TABLE 1:** Results of 30‐m sprint time and maximal ball velocity under control, caffeine, placebo, caffeine + static stretching, placebo + static stretching, and static stretching conditions.

Variables	CON	SS	CAF	PLA	CAF + SS	PLA + SS
Maximal ball velocity	100.8 ± 6.0	100.4 ± 6.2	101.5 ± 4.5^a^	99.6 ± 6.1	100.6 ± 5.5	100.2 ± 4.8
30‐m sprint time	4.36 ± 0.19^b^ ^,^ ^c^	4.37 ± 0.18^b^ ^,^ ^c^	4.28 ± 0.17^d^ ^,^ ^a^ ^,^ ^e^ ^,^ ^f^	4.36 ± 0.19^b^	4.31 ± 0.19^f^	4.35 ± 0.2^b^ ^,^ ^c^ ^,^ ^e^

Abbreviations: CAF, caffeine only; CAF + SS, caffeine combined with static stretching; CON, control; PLA, placebo only; PLA + SS, placebo combined with static stretching; SS, static stretching only.

^a^ Significantly different to SS condition.

^b^ Significantly different to CAF condition.

^c^ Significantly different to CAF + SS condition.

^d^ Significantly different to CON condition.

^e^ Significantly different to PLA condition.

^f^ Significantly different to PLA + SS condition.

A repeated measures ANOVA showed no significant differences across the conditions for maximal ball velocity (*F*(5, 75) = 1.11, *p* = 0.36, *η* ^2^_p_ = 0.069). However, pairwise comparisons revealed a statistically significant difference between the CAF condition (101.5 km·h^−1^) and the SS condition (99.6 km·h^−1^; *p* = 0.015; ES: 0.69; Table 1).

### 3.2 Individual Responses

When examining individual response distributions, the caffeine condition (*n* = 11) in the 30‐m sprint test showed improvements in 4 more participants compared with the CAF + SS condition (*n* = 7), whereas only 2 participants exhibited performance decrements in both conditions. Moreover, the number of participants who showed decrements in 30‐m sprint performance was noticeably higher in the other conditions (*n* = 6–8) compared with caffeine (*n* = 2) and CAF + SS (*n* = 2).

Maximal ball velocity results presented a different pattern from sprint performance. In maximal ball velocity, the number of participants showing improvements was similar across all conditions (*n* = 4–6). However, the caffeine condition (*n* = 2) had fewer participants with performance decrements compared with the other conditions (*n* = 4–6).

## 4 Discussion

This study aimed to investigate the independent and combined effects of CAF and SS on maximal ball velocity and 30‐m sprint performance. Our findings indicate that SS did not directly impair either maximal ball velocity or sprint performance, whereas CAF ingestion significantly enhanced sprint performance (Table 1). Moreover, the combination of CAF and SS was less effective than CAF alone in enhancing performance. Notably, a 2.2% reduction in 30‐m sprint time was observed following CAF ingestion. This result aligns with previously reported reductions of 1%–3% in sprint times across various sports disciplines. For instance, in trained sprinters, a 6 mg·kg^−1^ dose of CAF reduced 100‐m sprint times by an average of 0.14 s (∼1.2%) compared to PLA. The same study reported that increased acceleration during the 0–10 m and 10–20 m segments contributed to more explosive propulsion in the initial phase of the sprint. Furthermore, improvements in the first 60 m were directly associated with overall enhancements in 100‐m sprint performance. Similarly, in futsal and other team sports, CAF has been shown to enhance single and repeated sprint performance over distances of up to 40 m (López‐Samanes et al. 2021; Muñoz et al. 2020; Stojanović et al. 2019; Carr et al. 2008; Evans et al. 2018; Glaister et al. 2008; H. Mor et al. 2025). The ergogenic effect of caffeine has also been observed not only in running‐based sprints but in swimming sprints as well. For example, Acar et al. (2024) reported that ingestion of 6 mg·kg^−1^ of caffeine improved 25 and 50 m freestyle sprint performance in female swimmers, although it did not produce a significant effect on vertical jump performance. These findings indicate that CAF positively influences sprint performance, although its effectiveness does not extend equally across all performance outcomes.

In our study, the lack of an effect of CAF on maximal ball velocity suggests that caffeine may be limited in tasks requiring technical and multifactorial skills. Similarly, the literature has consistently reported that, although CAF provides benefits in sprint, jump, agility, and reaction time, it does not produce significant changes in maximal ball velocity. Indeed, H. Mor et al. (2025) in female soccer players, López‐Samanes et al. (2021) in futsal players, and A. Mor et al. (2024) in male soccer players observed improvements in sprint or other performance measures, but none found an effect on maximal ball velocity. Therefore, although caffeine demonstrates ergogenic effects in various performance tests, it appears to be consistently ineffective with respect to maximal ball velocity. In technical performance tests such as shooting, multiple factors, including balance, coordination, timing, and motor control, come into play and may mask the potential effects of CAF.

Although CAF is one of the few ergogenic aids with well‐established performance‐enhancing effects, whether these benefits manifest, and to what extent, may depend on the specific nature of the performance test. Although CAF ingestion significantly improved sprint time in our study, its lack of effect on maximal ball velocity may be attributed to the technical and multifactorial nature of this test variable (Yildirim et al. 2023). To better understand these varying effects of CAF, it is important to consider its physiological and neurological mechanisms. CAF acts as an adenosine receptor antagonist, blocking the inhibitory effects of adenosine on the central nervous system (CNS). This leads to increased neuronal activity and, consequently, enhanced alertness and concentration. When adenosine is inhibited, the release of neurotransmitters such as dopamine and norepinephrine increases, which in turn facilitates voluntary muscle activation by enhancing motor unit firing rates and sustaining neuro‐excitability (Fredholm et al. 1999; de Kivit et al. 2013; J. K. Davis and Green 2009).

Another key intervention examined in our study was SS, which can produce varying effects on performance, particularly in tests requiring short‐distance sprinting and explosive power. Behm et al. (2016) reported an average performance decline of 1.3% in strength‐ and speed‐related activities following SS but noted that this reduction was not sufficient to justify the removal of SS from warm‐up routines. Similarly, in our study, we observed a nonsignificant 1.2% decrease in maximal ball velocity and virtually no change in sprint performance under the SS condition compared to CON. Other findings in the literature also demonstrate the limited or inconsistent impact of SS. For example, Little and Williams (2006) reported that short‐duration SS (5 × 30 s, rest: 20 s) did not impair sprint or agility performance in professional football players and even led to improvements in 10 m acceleration and flying 20 m sprint times. Similarly, Hernández‐Martínez et al. (2023) found that SS (4 × 2 × 30 s with 45‐s rest intervals) had no significant effect on jump height, sprint speed (10, 20, 30 m), or instep kicking performance in young football players. On the other hand, some studies have reported contrasting results. Gelen (2010) observed significant performance decrements in 30 m sprint time, penalty kick ball velocity, and slalom dribbling performance following SS (5 × 2 × 20 s with 10‐s rest intervals) in professional football players. Amiri‐Khorasani and Ferdinands (2014) found conditioning‐dependent responses to SS, with maximal ball velocity during an instep kick decreasing by 4.5% in a low‐fitness group and by 3.7% in a high‐fitness group of young football players. Avloniti et al. (2016), who investigated the effects of different stretching durations, showed that 15‐ and 20‐s SS protocols improved 10 and 20 m sprint performance by 2.8%–3.2%, whereas shorter or longer durations did not lead to significant changes. Furthermore, participants in the moderate‐performance group improved their 10 and 20 m sprint times by 4.2% and 4.1%, respectively, under the 15‐ and 20‐s protocols, whereas those in the high‐performance group showed no change under any SS condition compared to CON.

**FIGURE 2:**
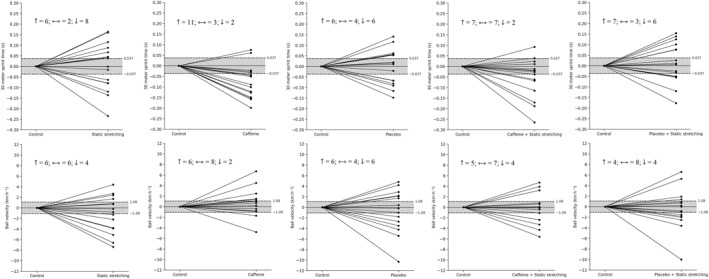
Individual performance changes in 30‐m sprint time (top row) and maximal ball velocity (bottom row) under different experimental conditions compared to the control condition. The dashed lines represent the smallest worthwhile change (SWC) thresholds for sprint time (± 0.037 s) and maximal ball velocity (± 1.08 km·h^−1^), with the shaded area indicating the “no meaningful change” zone. Values above the upper dashed line indicate a performance decrement in 30‐m sprint time (i.e., slower sprint times) and a performance improvement in maximal ball velocity (i.e., higher maximal ball velocity). Values below the lower dashed line indicate a performance improvement in 30‐m sprint time (i.e., faster sprint times) and a performance decrement in maximal ball velocity (i.e., lower maximal ball velocity). Each line connects an individual's performance in the control condition (left side of each plot) to their performance in the respective experimental condition. Some lines appear overlapped because several participants had identical or very similar values, which resulted in overlapping trajectories.

This variability in findings is also reflected in systematic reviews and meta‐analyses. For instance, Li et al. (2023) reported that SS negatively affected 20 and 30 m sprint performance compared to CON but had no significant effect on the vertical jump height. In contrast, Warneke and Lohmann (2024) suggested that SS protocols lasting no more than 60 s did not impair sprint or jump performance and might even lead to slight improvements. These conflicting findings may be attributed to methodological differences, including the duration, type, and targeted muscle groups of the SS protocols; individual participant factors such as age, training history, and fitness level; and the specific performance tests employed (Chaabene et al. 2019; Franco et al. 2008; Oshita et al. 2016; Avloniti et al. 2016). Moreover, contradictory results in meta‐analyses may stem from methodological discrepancies or publication bias, both of which can affect the reliability of the conclusions (Ma et al. 2021). Due to these inconsistencies in the literature, many studies suggest that the effects of SS on explosive performance still require definitive validation. However, such validations should be based on individualized assessments. Even participants of the same sex, with similar training backgrounds and performance levels, may exhibit different acute responses to the same SS protocols. Indeed, our sample group and individual data support this observation. Although our participants' comparable training backgrounds and the narrow dispersion in control performance (Coefficient of Variation: 4.46% for the 30 m sprint and 5.94% for maximal ball velocity) indicate the homogeneity of the sample, individual responses still varied. As shown in Figure 2 and Table 2, following SS, 50% of the participants exhibited a decline in 30 m sprint performance and 44% showed a reduction in maximal ball velocity performance; in contrast, 38% demonstrated improvements in sprint performance and 25% in maximal ball velocity. These findings indicate that the effects of SS may vary not only across individuals but also depending on the specific performance variable assessed.

**TABLE 2:** Individual expectations for caffeine or placebo, and performance responses in 30‐m sprint time and maximal ball velocity under caffeine, placebo, placebo + static stretching, and caffeine + static stretching conditions compared to control conditions.

		Expectation					Maximum ball velocity						30‐m sprint				
Part. No		CAF	PLA	CAF + SS	PLA + STA		SS	CAF	PLA	CAF + SS	PLA + SS		SS	CAF	PLA	CAF + SS	PLA + SS
1		CAF	PLA	CAF	PLA		↓	⟷	↓	↓	↓		↑	↑	↑	↑	↑
2		CAF	CAF	CAF	CAF		⟷	⟷	↑	↑	⟷		↑	↑	↑	↑	↑
3		CAF	PLA	CAF	PLA		↓	⟷	↓	↓	⟷		↓	↑	⟷	↑	⟷
4		PLA	CAF	PLA	CAF		⟷	⟷	↓	⟷	↑		↓	↓	⟷	↓	⟷
5		PLA	CAF	PLA	CAF		⟷	⟷	⟷	↓	⟷		↑	↑	↓	⟷	↑
6		CAF	PLA	PLA	CAF		↑	↑	↓	↑	↑		↓	⟷	⟷	↓	↓
7		PLA	PLA	CAF	PLA		↓	↑	↑	⟷	⟷		↓	↑	↓	⟷	↓
8		CAF	PLA	CAF	CAF		↑	↑	↑	↑	↑		↓	↓	↓	⟷	↓
9		CAF	PLA	PLA	PLA		↓	⟷	↑	⟷	↓		↓	↑	↑	↑	⟷
10		CAF	CAF	CAF	PLA		⟷	↓	↓	⟷	↓		↑	↑	⟷	↑	↑
11		PLA	CAF	PLA	CAF		⟷	⟷	⟷	⟷	⟷		⟷	⟷	↓	⟷	↓
12		CAF	CAF	PLA	CAF		↑	↑	↑	↓	⟷		↑	↑	↓	⟷	↓
13		PLA	CAF	PLA	PLA		⟷	↑	⟷	⟷	↑		↑	⟷	↑	⟷	↑
14		CAF	PLA	PLA	CAF		↓	↓	↓	↓	⟷		⟷	↑	↑	↑	↑
15		PLA	PLA	CAF	CAF		↓	⟷	⟷	⟷	↓		↓	↑	↑	↑	↑
16		CAF	PLA	PLA	PLA		↑	↑	↑	↑	⟷		↓	↑	↓	⟷	↓
Total	CAF	10	7	7	9	↑	6	6	6	5	4	↑	6	11	6	7	7
	PLA	6	9	9	7	⟷	6	8	4	7	8	⟷	2	3	4	7	3
						↓	4	2	6	4	4	↓	8	2	6	2	6

*Note:* Based on the SWC, maximal ball velocity and 30‐m sprint test results were classified as performance improvement (↑), no effect (⟷), or performance decrement (↓).

Abbreviations: CAF, caffeine only; CAF + SS, caffeine combined with static stretching; PLA, placebo only; PLA + SS, placebo combined with static stretching; SS, static stretching only.

This variability in the literature also highlights the need to consider not only the standalone effects of SS but also its potential to diminish the efficacy of other ergogenic aids, such as CAF, when used in combination. In our study, although SS alone did not significantly impair or enhance sprint performance, its combination with CAF resulted in a smaller performance benefit compared to CAF alone. Comparisons across other experimental conditions further suggest that SS may slightly blunt the performance‐enhancing effects of CAF. Although sprint performance in the SS condition was significantly lower than in the CAF condition, no significant difference was observed between the CAF and CAF + SS conditions. However, the fact that CAF alone produced superior sprint performance compared to the CAF + SS condition suggests a possible attenuating effect of SS on CAF's ergogenic potential. When comparing the CON and CAF conditions, we observed a statistically significant difference (*p* = 0.002) with a large effect size (ES = 0.94). In contrast, the CAF + SS condition yielded only a borderline significant difference compared to CON (*p* = 0.043), with a moderate effect size (ES = 0.54). These results further support the notion that SS may partially reduce the performance‐enhancing effects of CAF. This trend is also evident in our individual‐level data: when compared to the CON condition, 69% of participants (11 out of 16) in the CAF condition and 44% (7 out of 16) in the CAF + SS condition demonstrated improvements in sprint time exceeding the smallest worthwhile change. These findings provide additional support for the notion that the combination of CAF and SS yields a smaller performance benefit than CAF alone.

The evidence base is still in its early stages; however, data from the limited number of available studies (Farney et al. 2019; Çabuk et al. 2025) suggest that SS partially reduces the performance gains expected with CAF. In the study by Farney et al. (2019), participants experienced an approximate 7.5% decrease in 1RM knee flexion performance following an SS protocol, regardless of whether CAF or PLA was consumed, compared to the no‐stretching condition. These results suggest that SS may attenuate the ergogenic effects of CAF. In contrast, Çabuk et al. (2025) found that the performance decrements induced by SS were mitigated when CAF was consumed, compared to SS alone or PLA combined with SS. However, they still reported a partial attenuation of CAF's positive effects when combined with SS. According to Çabuk et al. (2025), although no statistically significant differences were observed between the CAF and CAF + SS conditions in peak power output average power output, minimum power, or time to peak power, the CAF group demonstrated higher mechanical power outputs by 3.8% (ES = 0.58), 1.4% (ES = 0.60), and 2.6% (ES = 0.29), respectively, and reached peak power 13% faster (ES = 0.14).

These findings are consistent with our current results. Although we did not observe statistically significant differences between the CAF and CAF + SS conditions in either maximal ball velocity or sprint time, participants in the CAF group exhibited better performance, by 0.9% (ES = 0.37) in maximal ball velocity and 0.7% (ES = 0.35) in sprint time. Moreover, in the findings of Çabuk et al. (2025), the CAF condition resulted in significantly higher maximal cadence values (∼3%) compared to the CAF + SS condition (ES = 0.66), further supporting the idea that SS may attenuate CAF's ergogenic effects. Additionally, the effect sizes between the CON and CAF conditions for peak power output, average power output, and maximal cadence (ES = 0.72–0.93) were considerably greater than those between the CON and CAF + SS conditions (ES = 0.18–0.34), suggesting a limiting effect of SS on CAF's performance‐enhancing potential. Furthermore, their study found a significant performance difference between the CON and CAF conditions, whereas performance values in the CON and CAF + SS conditions were similar, again reinforcing this interpretation. In our study, both the CAF and CAF + SS conditions led to significant improvements in sprint performance compared to the CON. However, the marginal significance observed between the CAF + SS and CON conditions suggests that SS may have partially blunted the ergogenic effect of CAF. Although the physiological mechanisms underlying such interactions are not yet fully understood, current evidence points toward the involvement of the central nervous system (CNS). As noted by Behm et al. (2016), a substantial portion of the literature suggests that performance decrements following SS are most likely due to reductions in CNS activity (Behm et al. 2016; Chaabene et al. 2019; Palmer et al. 2019; Trajano et al. 2013). SS may reduce muscle spindle sensitivity and suppress reflex pathways, ultimately decreasing *α*‐motor neuron excitability and lowering motor unit activation (Trajano et al. 2013; Palmer et al. 2019).

Although SS alone did not result in a meaningful decline in sprint performance in our study, it may have interfered with the full manifestation of CAF's stimulating effects on the CNS. Under normal circumstances, CAF enhances voluntary muscle activation by blocking adenosine receptors and increasing the release of neurotransmitters such as dopamine and norepinephrine, thereby elevating motor unit firing rates and sustaining neuro‐excitability (Fredholm et al. 1999; de Kivit et al. 2013; J. K. Davis and Green 2009). However, these effects may not have fully translated in the post‐SS environment. Additionally, the ergogenic effects of CAF are not exclusively physiological and may also be influenced by positive or negative expectancy effects (Beedie et al. 2007). To address this, a double‐blind, placebo‐controlled design was implemented in our study to ensure that participants were unaware of their assigned condition, thereby minimizing potential expectancy effects. Participants were also asked before testing what they believed they had consumed, which allowed us to interpret both the actual and perceived effects. In the CAF and CAF + SS conditions, 63% and 44% of participants, respectively, believed they had ingested CAF (Table 2). In contrast, 44% and 56% of participants in the PLA and PLA + SS conditions, respectively, believed they had received CAF (Table 2). These findings indicate a relatively even distribution of perceived CAF intake across all four conditions, suggesting that expectancy effects were either eliminated or substantially reduced. Thus, our findings suggest that although CAF had no effect on maximal ball velocity, its ergogenic effect on sprint performance was likely not driven by belief or perception, but rather by the specific nature of the performance test. Moreover, although 70% and 44% of participants showed performance improvements in the sprint test under the CAF and CAF + SS conditions, respectively, only 38% and 31% showed improvements in the maximal ball velocity test under the same conditions (Table 2 and Figure 2). This indicates that individual responses may also play a role. These differences could be influenced by factors such as genotype variations, individual sensitivity to CAF, the dosage administered, or the time required for CAF to reach peak plasma levels (Pickering and Kiely 2018).

Although the effects of CAF on performance may be influenced by these variables, the International Olympic Committee (2018), in its consensus statement on dietary supplements, recognizes CAF as one of the few supplements with well‐documented performance‐enhancing effects (Maughan et al. 2018). On an individual level, it should also be noted that even minor changes in performance may yield significant outcomes in certain sports. For example, in the 2024 Paris Olympic Games 100‐m final, the time difference between the gold medalist and the fourth‐place finisher was only 0.03 s (Çabuk et al. 2025). This example illustrates how interventions perceived as having small effects, such as SS or CAF, or their combination, can be decisive in high‐performance contexts. Therefore, the interaction effects of such strategies, whether applied individually or in combination, should be carefully considered, particularly in competitive settings.

### 4.1 Limitations

Several limitations should be acknowledged. First, the performance tests were conducted indoors on a wooden surface; performing the same tests on a natural soccer field (grass) might have produced different outcomes, as surface properties can influence spatial orientation, joint loading, muscle activation, and technical execution (e.g., stabilization of the support leg, mechanics of the kicking leg, and sprint running). Second, no specific recovery strategies were applied or monitored after the testing sessions, which may have affected subsequent performance or participant readiness. Third, dietary intake was not strictly controlled beyond caffeine and alcohol restrictions, which could have introduced variability in nutritional status. Fourth, the absence of baseline measurements prior to each condition may have made it more difficult to account for daily fluctuations in performance (e.g., fatigue, motivation, nutrition, or recovery status). However, this limitation was intentionally considered in the study design. The static stretching protocol lasted approximately 22 min, which could have altered the timing of peak plasma caffeine concentration, particularly in the CAF + SS and PLA + SS conditions. In addition, baseline testing might have induced a small but meaningful post‐activation performance enhancement effect, potentially confounding the results. Fifth, the relatively small sample size (*n* = 16) reduced statistical power and limited the generalizability of the findings. Given the subtle nature of the effects tested (e.g., the attenuation of caffeine's ergogenic benefits when combined with static stretching), individual variability and daily fluctuations may have had a more pronounced influence on the outcomes. Future research with larger sample sizes is warranted to enhance the reliability and external validity of these findings.

Finally, the study included only male participants, which may limit the generalizability of the results. This decision was made to reduce within‐sample variability and to ensure a more homogeneous participant group. Conflicting evidence in the literature regarding sex‐specific responses to caffeine also contributed to this methodological choice. Although some studies have reported significant sex‐related differences, others have not. Nevertheless, the exclusion of female participants remains a limitation that restricts the broader applicability of the findings.

## 5 Conclusion

This study demonstrates that CAF ingestion significantly enhances 30‐m sprint performance, whereas SS alone has no meaningful effect on either maximal ball velocity or sprint performance. However, when SS and CAF are applied together, SS appears to partially diminish the ergogenic benefits of CAF. Notably, the absence of any effect of CAF on maximal ball velocity suggests that the efficacy of this supplement may vary depending on the complexity and technical demands of the task. Moreover, the observation of different responses among participants highlights the role of individual variability and underscores the importance of personalized approaches in optimizing performance. Although CAF remains one of the few supplements with consistently supported performance‐enhancing effects, its interaction with SS should be carefully considered, particularly in elite settings where even marginal gains can be decisive.

Future research should include both male and female athletes with different training backgrounds, conduct tests on various playing surfaces, and incorporate dietary monitoring and recovery protocols. Additionally, exploring the combined effects of CAF intake with various stretching modalities, such as dynamic, ballistic, and proprioceptive neuromuscular facilitation, or integrated stretching protocols could offer valuable guidance for optimizing warm‐up strategies to enhance athletic performance.

## Author Contributions

**Refik Çabuk:** data collection, conception or design of work, data analysis and interpretation, drafting the article, critical revision of the article. **İzzet İslamoğlu:** data collection, drafting the article, critical revision of the article. **Onur Demirarar:** conception or design of work, critical revision of the article. **Faruk Albay:** methodology, investigation, validation, writing ‒ review and editing. **Yıldırım Kayacan:** conception or design of work, drafting the article, critical revision of the article. All authors read and approved the final manuscript.

## Ethics Statement

The study was approved by the Institutional Review Board of the Gendarmerie and Coast Guard Academy (Ethics approval number: E‐97703210‐050.99‐37067511)

## Conflicts of Interest

The authors declare no conflicts of interest.

## Data Availability

Data generated and/or analyzed during this study are available from the corresponding author upon reasonable request.
